# Review on Medical Implantable Antenna Technology and Imminent Research Challenges

**DOI:** 10.3390/s21093163

**Published:** 2021-05-02

**Authors:** Md Mohiuddin Soliman, Muhammad E. H. Chowdhury, Amith Khandakar, Mohammad Tariqul Islam, Yazan Qiblawey, Farayi Musharavati, Erfan Zal Nezhad

**Affiliations:** 1 Department of Electrical, Electronic & Systems Engineering, Universiti Kebangsaan Malaysia, Bangi, Selangor 43600, Malaysia; p108446@siswa.ukm.edu.my (M.M.S.); tariqul@ukm.edu.my (M.T.I.); 2 Department of Electrical Engineering, Qatar University, Doha 2713, Qatar; amitk@qu.edu.qa (A.K.); yazan.qiblawey@qu.edu.qa (Y.Q.); 3 Mechanical & Industrial Engineering Department, Qatar University, Doha 2713, Qatar; 4 Department of Biomedical Engineering, University of Texas at San Antonio, San Antonio, TX 78249, USA; erfan.zalnezhad@utsa.edu

**Keywords:** medical implantable antenna, antenna design, specific absorption rate, implant fabrication, biocompatibility

## Abstract

Implantable antennas are mandatory to transfer data from implants to the external world wirelessly. Smart implants can be used to monitor and diagnose the medical conditions of the patient. The dispersion of the dielectric constant of the tissues and variability of organ structures of the human body absorb most of the antenna radiation. Consequently, implanting an antenna inside the human body is a very challenging task. The design of the antenna is required to fulfill several conditions, such as miniaturization of the antenna dimension, biocompatibility, the satisfaction of the Specific Absorption Rate (SAR), and efficient radiation characteristics. The asymmetric hostile human body environment makes implant antenna technology even more challenging. This paper aims to summarize the recent implantable antenna technologies for medical applications and highlight the major research challenges. Also, it highlights the required technology and the frequency band, and the factors that can affect the radio frequency propagation through human body tissue. It includes a demonstration of a parametric literature investigation of the implantable antennas developed. Furthermore, fabrication and implantation methods of the antenna inside the human body are summarized elaborately. This extensive summary of the medical implantable antenna technology will help in understanding the prospects and challenges of this technology.

## 1. Introduction

Significant progress has been observed over decades in medical applications with the development of modern technology. The use of communication tools has brought unimaginable success in the medical sector, which saved millions of people from life-threatening diseases such as congestive cardiac failure, cancer, and diabetes [1,2,3,4]. In the past, the implanted devices were powered and monitored by an external station via wired connectors, which poses a risk and hazard to the human body and requires unavoidable surgical procedures. Implantable antenna technology has reduced the risk and hazard which was associated with surgical procedures and made this suitable for medical applications [5]. In contemporary times, under provisional implant antenna technology, implantable wireless chips are being used to trace people or pets, to collect information from the body to external monitoring devices [6].

**Figure 1:**
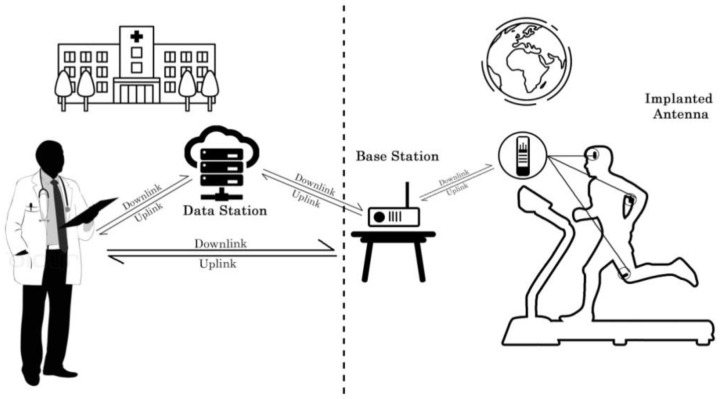
A typical implantable antenna system.

A typical implant antenna system is shown in Figure 1, where the implanted transceiver positioned at various human organs interconnect with the external base station and is logged at a data station and can also be monitored by the respective medical personnel using a wireless interface [7,8]. An implanted antenna communication system contains three parts: antenna, modulator, and demodulator. The implanted antenna communicates with the external source via uplink and downlink. The uplink connects the implant antenna to the external device while the external device communicates with the implant antenna via downlink [8]. Radio wave propagation through the heterogeneous lossy tissue inside the human body causes absorption of most of the antenna radiation [9]. Consequently, the inhomogeneous organ structure leads to impedance mismatch, which makes the radiation characteristic inefficient. Researchers are developing diverse antenna techniques to increase antenna efficiency [10,11]. The powering of the implant antenna system inside the human body is a major research challenge. Bulky battery size with limited battery life further complicates the system design [12]. Nowadays, wireless power transfer (WPT) to the implanted antenna is of great research interest [13,14,15,16]. WPT requires a proper selection of the frequency band, which is an important part of the implant antenna system for medical applications. The selected frequency band must avoid electromagnetic interference with the existing terrestrial frequency bands [17,18,19]. Furthermore, the verification and fabrication of such miniature implant antennas inside the human organ are incredibly challenging due to the limited accredited animal laboratories, along with health safety issues [10,20,21]. This work aims to summarize the recent works published about implant antenna design, implant antenna design factors, diverse fabrication technology, and future research challenges.

The rest of this manuscript is organized as follows. Section 2 summarizes the radio frequency spectrum allocation for implanted medical devices by multiple regulatory institutions. Factors that affect the propagation of radio frequency through the human body tissues are discussed in Section 3. Section 4 describes a parametric literature investigation on the implanted antennas. In Section 5, fabrication and implantation methods of the antenna inside the human body are reviewed in detail. The limitations of the different implantable antennas are discussed and future research challenges are highlighted in Section 6. Finally, a summary is provided in Section 7.

## 2. Radiofrequency Spectrum Allocation

The radio spectrum allocation for wireless medical applications varies between regions maintained by different accredited regulatory organizations. Radio spectrum allocation in each region is also classified depending on license radio spectrum approaches for unique medical applications and shared radio spectrum with other applications.

### 2.1. Radio Frequency Spectrum Allocations in the United States

The complete wireless medical device system in the United States is regulated by three authorized federal agencies: the Federal Communication Commission (FCC), the Food and Drug Administration (FDA), and the Centers for Medicare and Medicaid Services (CMS). FCC is responsible for allocating radio frequency spectrum while FDA maintains the regulations of patient safety, and CMS regulates the costing system [17,18]. The classification of frequency spectrum allocations can be divided into short-range and long-range classes, according to FCC, as summarized below:

#### 2.1.1. Short Range Wireless Medical Devices

The frequency spectrum allocation for potential short-range wireless devices regulated by FCC is summarized below:

▪ Inductive Implant—The frequency spectrum below 100 kHz is allocated for inductive implant medical devices that can transport a small amount of data, and when continuous data transmission is not required [17,18].
▪ Medical Device Radio Communication Service (MDRC)—The frequency range 401–402 and 405–406 MHz are allocated for wearable medical applications. The channel bandwidth limit is 100 kHz for the bands of 401–402 and 405–406 MHz. On the other hand, 300 kHz is permitted for the band of 402–405 MHz [17,18].
▪ Ultra-wide Band (UWB)—UWB is an emerging wireless band for medical applications in the band of 3.1–10.6 GHz. Wireless medical devices that are operating in UWB provide higher data transfer rate of 1 Gbps for a short range of communication (<1 m).
▪ Medical Micropower Networks (MMNs)—FCC assigned a 24 MHz frequency spectrum in 413–457 MHz frequency band for implant medical device applications. The frequency spectrum is divided into four segments: 413–419, 426–432, 438–444, and 451–457 MHz [18].
▪ Medical Body Area Networks (MBANs)—According to the General Electronic Health care (GEHC) proposal, FCC allocated a frequency spectrum in the band of 2360–2400 MHz to monitor human body conditions using medical body area networks [18].
▪ Wi-Fi, Bluetooth, and Zigbee—FCC declared the frequency spectrum for Wi-Fi, Bluetooth, and ZigBee in the frequency bands of 902–928, 2400–2483.5, and 5725–5850 MHz, respectively. These frequency spectrums can be used for short-range digital modulation communication applicable for implant medical [18].

#### 2.1.2. Long Range Wireless Medical Devices

Long-range communication refers to communication between 30 m to 10 km distance for medical applications. The frequency spectrum allocated for diverse wireless medical applications is summarized below [18].

▪ Wireless Medical Telemetry Services (WMTS)—FCC allocated at 13 MHz spectrum for WMTS in the frequency bands of 608–614, 1395–1400, and 1427–1429 MHz. WMTS devices are used for monitoring the patient’s health condition via a bi-directional wireless link [18].
▪ World Interoperability for Microwave Access (WiMAX)—The frequency spectrum allocated for WiMAX in the frequency band of 2.5 GHz, according to the IEEE 802.16. WiMAX can transmit approximately 70 Mbps, which is very efficient in data transfer, it is used to transfer information from an ambulance to the hospital [18].

### 2.2. Radio Frequency Spectrum Allocations in Europe

The radio frequency spectrum allocation in Europe recommended by the Electronic Communication Committee (ECC) is summarized below [19]:

#### 2.2.1. Active Medical Implants and Associated Peripherals 

According to the ECC recommendations, the frequency spectrum of 9–315 kHz is utilized for active medical implant devices in an ultra-low-power range. Moreover, the frequency band of 30–37.5 MHz is used for blood pressure measurement devices in the ultra-low-power range. Finally, the frequency band of 2483.5–2500 MHz is allocated for implanted medical devices.

#### 2.2.2. Medical Data Acquisition Band

**Figure 2:**
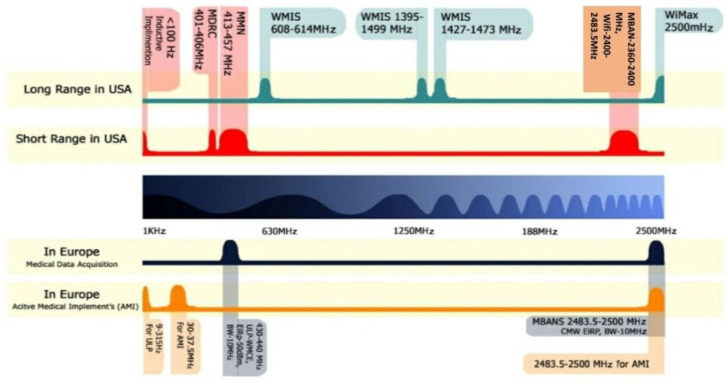
Radiofrequency spectrum allocation for wireless medical applications in the USA and Europe.

This frequency spectrum allocation is designated for non-implantable wearable medical devices used for monitoring the patient’s health condition remotely. This band consists of two sub-bands:

▪ Ultra-Low Power Wireless Medical Capsule Endoscopy (ULP-WMCE)—430–440 MHz frequency band is allocated for the wearable ULP-WMCE applications. The effective channel bandwidth is 10 MHz with Equivalent Isotropically Radiated Power (EIRP) −50 dBm.
▪ Medical Body Area Network System (MBANS)—The frequency spectrum of 2483.5–2500 MHz allocated for MBANS is utilized for patient monitoring devices in indoor communications. The effective isotropic radiated power set for MBANS devices is 1 mW for a channel bandwidth of 3 MHz. In Figure 2, spectrum allocation for wireless medical applications in the United States and Europe is shown.

## 3. Factors that Affect the Design of an Implantable Antenna

The development of the human body implantable antenna faces several challenges. Miniaturization of the antenna dimensions owing to the reduction of effective wavelengths needs to be upheld. To ensure patient safety, some factors need to be considered during the design phase. First, the implant antenna needs to be biocompatible, and the specific absorption rate (SAR) must be maintained within the specified limit. This section describes the factors that affect the implant antenna design.

### 3.1. Impact of Tissues Diversification

Radio wave propagation through the human body is a more complex, rather than free space, wave propagation due to the lossy property of the human tissue, which causes absorption of the radio wave, as illustrated below:

#### 3.1.1. Radio Wave Propagation in the Lossy Medium

**Figure 3:**
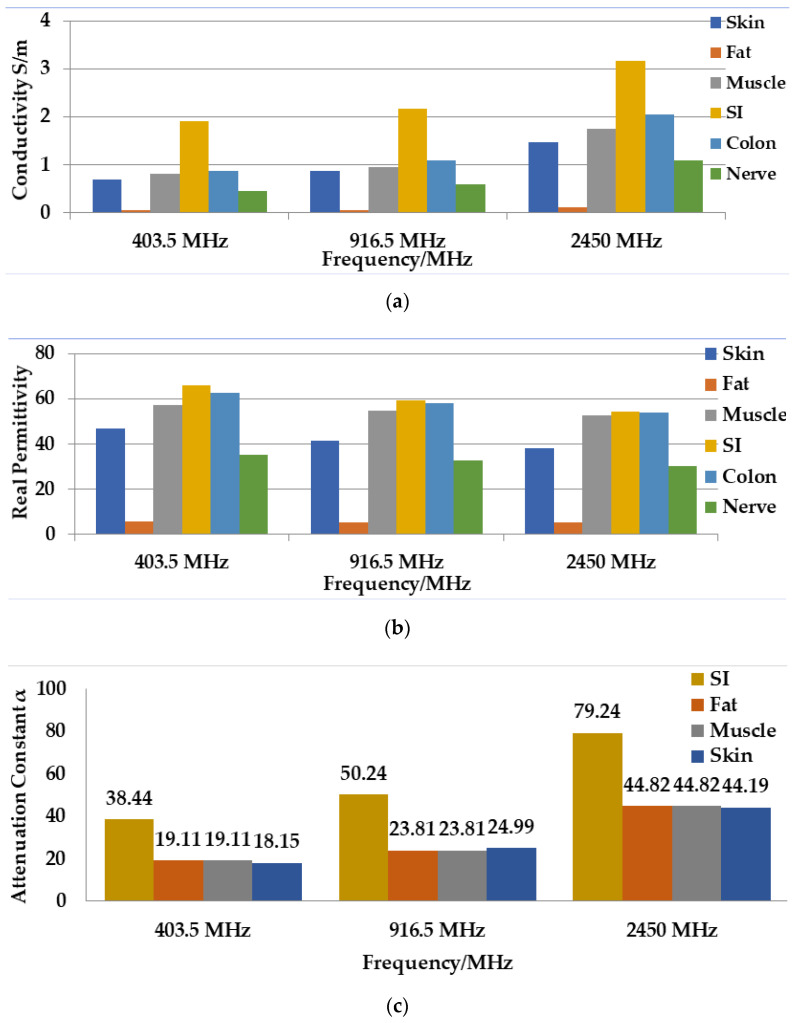

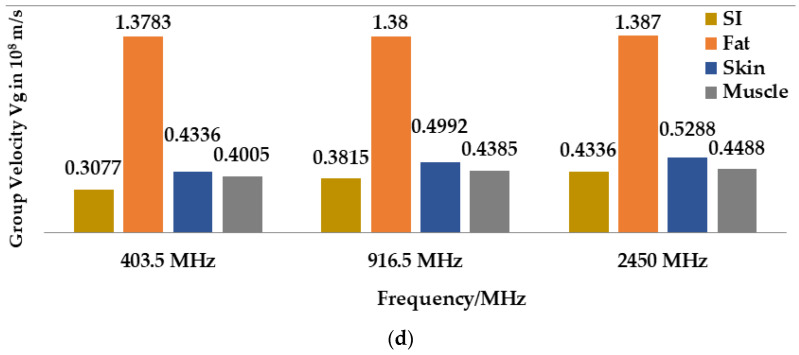
An illustration of conductivity (**a**), real permittivity (**b**), attenuation constant (**c**), and group velocity (**d**) for different frequency bands for biological tissues.

The radio waves are mainly characterized by the permeability, permittivity, and conductivity of the medium. An electromagnetic wave traveling in the Z-direction is defined by Equation (1) [22]:

(1) $$E\left(z\right)={\mathrm{Ee}}^{-\gamma z}$$

where E is the amplitude of the wave in the z-direction and γ is the complex propagation constant. The complex propagation constant γ is defined by Equation (2) [23]. As stated by Equation (1), the continued increment of the complex wave propagation value leads to attenuation of the electromagnetic wave inside the inhomogenous region:

(2) $$\gamma =j\omega \sqrt{\mu \varepsilon }$$

where $\mu =\mu_{r}\mu_{0}$ defines the permeability of the medium and $\mu =\mu_{0}$ for human tissues. However, the relative permittivity of the lossy medium provides complex characteristics due to the conductivity of the medium. The permittivity of the lossy medium is defined by Equation (3) [22].

(3) $$\varepsilon \left(\omega \right)=\varepsilon \prime \left(\omega \right)-j\varepsilon \prime \prime \left(\omega \right)={\varepsilon^{\prime }}_{r}\left(\omega \right)\varepsilon -j{\varepsilon^{\prime \prime }}_{r}\left(\omega \right)\varepsilon_{0}$$

where the real part of relative permittivity is defined by ε′ and the imaginary part is by ε″. The imaginary part is retrieved from Equations (4) and (5) [22].

(4) $${\varepsilon^{\prime \prime }}_{r}\left(\omega \right)=\frac{\sigma }{\omega \varepsilon_{0}}$$

(5) $$\tan\delta =\frac{{\varepsilon^{\prime \prime }}_{r}}{{\varepsilon^{\prime }}_{r}}$$

where σ is the conductivity of the medium and tanδ is the loss tangent, which is defined as the ratio of the imaginary part of the relative permittivity to the real part of the relative permittivity. Hence, according to Equation (4) the greater conductivity leads to a higher complex permittivity value, where the frequency increment causes a lower value of imaginary relative permittivity in a lossy medium. Consequently, the increment of loss tangent value makes the medium lossy. The loss tangent values for different biological tissues have been discussed in detail in [24]. Figure 3a,b demonstrates the data retrieved from the article [25] and it can be seen that different organs exhibit different conductivity and permittivity at different frequencies. Notably, the conductivity increases with the frequency (Figure 3a). Consequently, as can be observed in Equations (4) and (5), the conductivity increases with the loss-tangent values of the medium. Besides, real relative permittivity decreases with the increment of frequency, as confirmed by data in [25].

The complex propagation constant is derived by combining Equations (2) and (3).

(6) $$\gamma =j\omega \sqrt{\mu \varepsilon^{\prime }}\sqrt{1-j\frac{\sigma }{\omega \varepsilon \prime }}$$

However, the complex propagation constant γ is made up of attenuation constant, α (real part) and phase constant, β (imaginary part), which are as follows:

(7) $$\alpha =\omega \sqrt{\frac{\varepsilon_{0}\mu_{0\;}{\varepsilon^{\prime }}_{r}}{2}\left(\sqrt{1+{\left(\frac{\sigma }{\omega \varepsilon_{0}{\varepsilon^{\prime }}_{r}}\right)}^{2}}-1\right)}$$

(8) $$\beta =\omega \sqrt{\frac{\varepsilon_{0}\mu_{0\;}\varepsilon \prime_{r}}{2}\left(\sqrt{1+{\left(\frac{\sigma }{\omega \varepsilon_{0}{\varepsilon^{\prime }}_{r}}\right)}^{2}}+1\right)}$$

Figure 3c shows the value of the attenuation constant for different operating frequencies, as per data found in [25]. Notably, the small intestine (SI) tissue generated a larger value of attenuation constant. Besides, the value of the attenuation constant is increased with the increase in frequency and conductivity of the medium according to Equation (7).

In summary, the complex propagation constant relies on three parameters: permittivity, permeability, and conductivity. The increase in conductivity makes the medium lossy, and the radio waves are significantly attenuated in the lossy medium. Hence, electromagnetic wave attenuates with the increment of complex propagation constant value as stated by Equation (1).

#### 3.1.2. Propagation Speed inside the Human Body

The propagation speed of the radio wave decreases due to the complex inhomogeneous characteristics of human body tissue. Thus, the radio wave propagation speed depends on the permittivity and conductivity of the medium. The propagation speed in any medium contains phase ($V_{p}$) and group velocity $\left(V_{g}\right)$, as shown below [23,26]:

(9) $$V_{p}=\frac{\omega }{\beta }\;$$

(10) $$V_{g}=\frac{\partial \omega }{\partial \beta }$$

where ω is the angular frequency and β is the phase constant described in Equation (8). The propagation speed depends on the phase constant, and therefore it diminishes as the conductivity of the medium increases. As shown in [25], the group velocities, in contrast to different frequency bands for different biological tissues, are shown in Figure 3d. It is shown that the higher conductivity of the medium the greater the reduction in propagation speed. On the other hand, the propagation speed increases with the rise of frequency. Besides, the fat body tissue showed a higher $V_{g}$ compared to other mediums. In summary, it can be said that the propagation speed reduces due to the higher conductivity of biological tissues.

### 3.2. Impact on Effective Wavelength

As stated by Pozar et al. in [23], the effective wavelength in any medium is inversely proportional to phase constant β, as defined in Equation (12).

(11) $$\lambda =\frac{2\pi }{\beta }$$

The phase constant, β, is proportionally dependent on conductivity, σ. The increment of medium conductivity helps to reduce the effective wavelength λ, which consequently leads to the miniaturization of the antenna inside the human body.

### 3.3. Biocompatible Encapsulation of the Implantable Antenna

**Figure 4:**
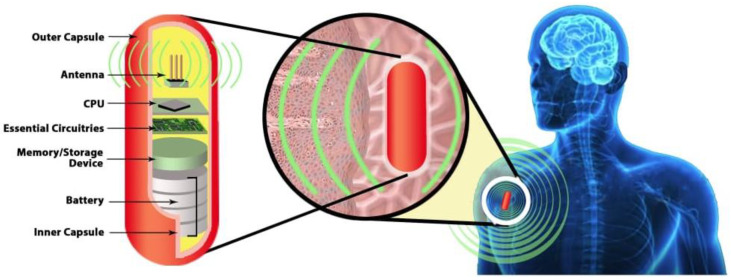
Encapsulation of Implantable Medical Antenna.

The biocompatibility of an implantable antenna is typically ensured by covering the antenna with an insulating material to restrain biological tissue from direct contact with the antenna. The conductivity of biological tissue creates a short circuit with the antenna patch, which is hazardous and will make the antenna useless. It was observed in the literature that two approaches are widely used to make the antenna biocompatible. Those are coating the implantable antenna with the layer of superstrate dielectric element (e.g., Teflon, Mackor, Ceramic alumina [27]) and encapsulating the implantable antenna with the low-loss dielectric element (e.g., Zirconia [28], polyether-ether ketone (PEEK) [29], Silastic Grade Elastomer [30,31]). Typical dielectric insulating materials are listed in the table given in [32]. An overall diagram on biocompatible encapsulation is shown in Figure 4, where antenna components were covered with biocompatible materials. A typical implantable system comprises of antenna for communication, electronics components such as CPU, memory for processing and storage, and finally, the battery to power up the implant.

### 3.4. Impact on Radiated Power and Efficiency

**Figure 5:**
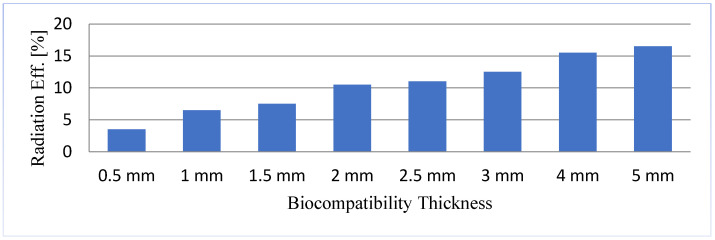
Comparison of radiation efficiency with respect to the thickness of biocompatible material.

The source power of an implanted antenna is the summation of radiated power and absorbed power [10].

(12) $$P_{\mathrm{source}}=P_{\mathrm{rad}}+P_{\mathrm{abs}}$$

where $P_{\mathrm{rad}}$ is the radiated power, and $P_{\mathrm{abs}}$ is the absorbed power by the surrounding lossy medium. Furthermore, the source power can be rewritten to the summation of radiated and absorbed power in the near and far-fields [10].

(13) $$P_{\mathrm{source}}=P_{\mathrm{rad}-\mathrm{FF}}+P_{\mathrm{rad}-\mathrm{NF}}+P_{\mathrm{abs}-\mathrm{FF}}+P_{\mathrm{abs}-\mathrm{NF}}$$

where $P_{\mathrm{rad}-\mathrm{FF}}$, $P_{\mathrm{rad}-\mathrm{NF}}$, $P_{\mathrm{abs}-\mathrm{FF}}$, and $P_{\mathrm{abs}-\mathrm{NF}}$ refer to the radiated and absorbed power in the near and far-fields. The outer boundary of the near field region and far field are commonly assumed to exist at a distance of $R<0.62\sqrt{D^{3}/}\lambda$ and $R<2D^{2}/\lambda$. where D is the antenna’s largest dimension and λ is the wavelength [33]. According to the antenna theory [23], the near field region is a reactive region for the lossless medium, where the antenna does not perform either radiation or absorption. On the other hand, in the lossy medium, the radiated radio wave causes strong coupling with the nearby lossy biological tissues. Therefore, the coupling of frequency leads to loss of radiated power, and this coupling is the primary concern of low radiation efficiency of an implantable antenna. Biocompatibility encapsulation using insulating materials plays a vital role in reducing the coupling with the nearest lossy environment. Figure 5 shows the relationship between the radiation efficiency and the thickness of biocompatible material. By increasing the thickness of biocompatible material (Superstate, $\epsilon_{r}=10.2$), the radiation efficiency can be increased, as stated by [10]. 

### 3.5. Requirement of Specific Absorption Rate (SAR)

The reduction of the specific absorption rate (SAR) is a significant research challenge in wireless implantable antennas for biomedical telemetry applications. The absorption of radiated power by the lossy biological tissues causes an issue in the surroundings of the implanted antenna and it is hazardous to the patient’s health. According to the FCC and ECC, the SAR value must be lower than 1.6 W/kg [34] and 2 W/10 kg [35], when 1 g and 10 g tissues are taken as standard.

### 3.6. Consideration in Powering System

Delivering power to the implanted antenna system is one of the major challenges for implantable antennas. Batteries are an inefficient solution for this application because they are short in time life, contain hazardous materials, and require a surgical operation to replace [36]. Besides, the power system must be lightweight and easy to fabricate to ensure the mobility of the patients. During the design of the power system, the energy level of the system has to comply with the regulations. Due to the limitations of the battery, significant research is now focusing on wireless power transmission, which will be covered in Section 5.

## 4. Summary of the Existing Implant Antenna Technology

This section highlights the summary of the different implant antennas. This section is divided into two sub-sections, where in the first sub-section, different antenna design techniques are discussed and in the second sub-sections, applications of implant antenna for different wireless applications are discussed.

### 4.1. Different Antenna Design Techniques 

Multiple implant antennas have been designed for different wireless medical applications. The developed implant antenna is categorized based on design aspects, such as miniaturization of antenna dimension, bandwidth enlargement technique, impedance matching stability technique, SAR reduction technique, directivity, increased efficiency, and biocompatibility technique.

#### 4.1.1. Miniaturization of Antenna Dimension

Radio wave propagation through the lossy medium having relatively large permittivity leads to the reduction of the effective wavelength. This increases the importance of the miniaturization of the antenna dimension. Several miniature antennas utilizing diverse design techniques will be discussed in the subsequent section.

**Figure 6:**
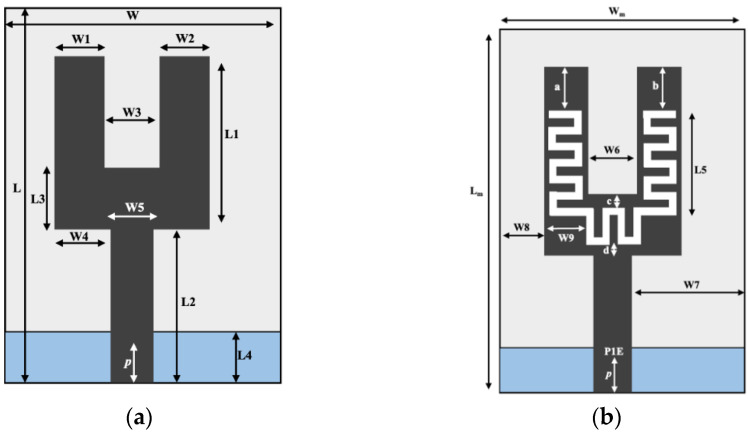
Implant antenna: without meandering slot (**a**) with meandering slot (**b**) [37].

**Figure 7:**
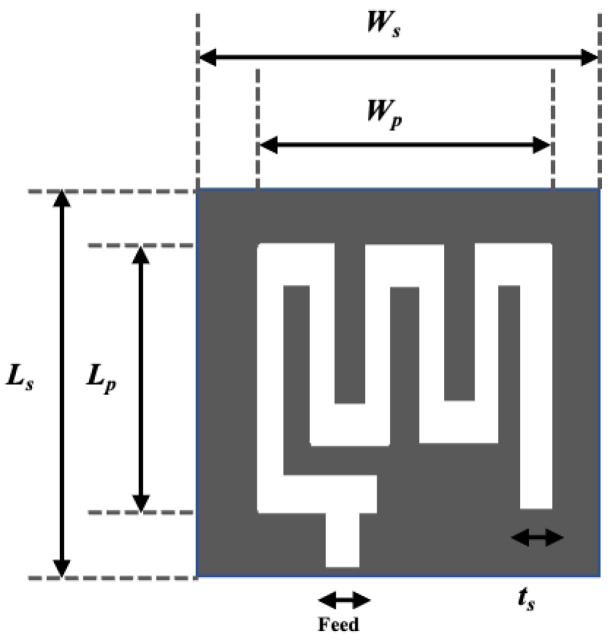
Meandered implant antenna proposed in [38].

**Figure 8:**
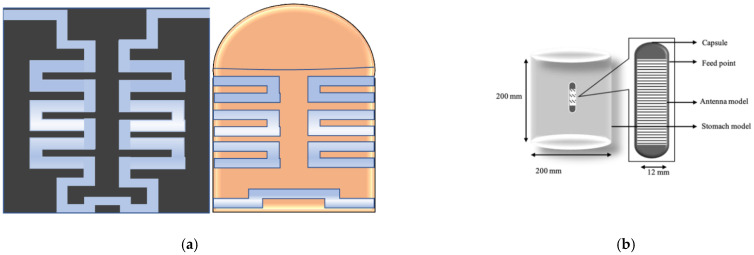
Geometry of capsule shape implant antenna (**a**) [39] and (**b**) [42].

In [37], a U-shape meandered slot antenna was proposed for implant medical applications operated at 2.45 GHz. It is shown in Figure 6 that the antenna dimension decreased to W × L (29 × 35 mm^2^) from W × L (33 × 40 mm^2^) after the meandered slots insertion. The insertion of meandered/spiraling slots on the radiating patch results in incrementing the current path on the same antenna dimension. This increment of the current path can be utilized to minimize the antenna dimension. The meandered slot’s insertion on the radiating patch resulted in the enlargement of bandwidth from 384 MHz to 396 MHz, and the S_11_ parameter value improved to −43.72 dB from −23.22 dB. The authors of [38] proposed a meandered slot-shaped implant antenna, which is suitable for the medical device radio communications service (MICS) frequency band (401–406 MHz) applications (Figure 7). It was reported that the substrate dimension is reduced by 36.85% in length and 40% in width and the antenna was examined in an inhomogeneous human tissue environment. Furthermore, a capsule shape meandered slot antenna is proposed in [39,40,41,42] for implant endoscopy applications. The geometry of the capsule-shaped implant antennas operated at the MICS band and UWB are shown in Figure 8. The implant antenna dimension is reduced to 5.5 × 10 mm^2^ (Rc × Lc) in [39] and 6 × 36.2 mm^2^ (Rc × Lc) in [42] for altering rectangular antenna design to capsule shape design. Moreover, cylindrical and circular shapes with meandered slot dual-band (401–406 MHz and 2.4 MHz) implant antennas were proposed in [43,44].

#### 4.1.2. Spiral Shape Radiating Patch

**Figure 9:**
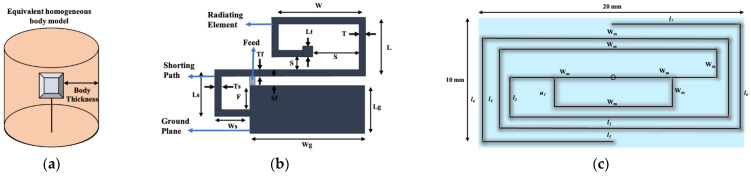
Geometry of Spiral shape implant antenna (**a**) [29], (**b**) [45], (**c**) [46].

A spiral shape radiating patch is a useful antenna dimension miniaturization technique employed in [29,45,46,47,48,49]. Figure 9a illustrates a spiral shape implant antenna operated at 225–427 MHz with an equivalent homogeneous body model. The spiral shape helps to miniaturize the antenna dimensions to 17 × 17 × 18 mm^3^ compared with other antennas given in [29]. Two spiral shape implant antennas to occupy a small area of 6 × 5 × 0.3 mm^3^ [45] and 20 × 10 × 1.63 mm^3^ [46] operated at 2.4 GHz and 405 MHz, respectively are shown in Figure 9b,c. Several miniaturized dimensions with the spiral shape Complementary Split Ring Resonator (CSRR)-loaded implant antenna was reported in [47,48,49] for MICS applications. 

**Figure 10:**
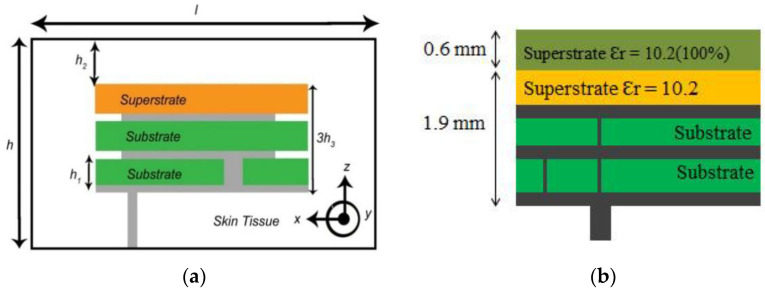
Stacking layer implant antenna employed in, (**a**) [50], (**b**) [51].

Antenna dimension can be miniaturized with the substrate material with higher relative permittivity suitable for wave propagation through the lossy medium. In the stacking patch layers technique, relative permittivity increases with the multi-layers dielectric substrate. Similarly, multi-layer dielectric element loading increases the current path, leading to a decrease of resonance frequency and the antenna dimension’s miniaturization. The stack patch implant antenna operated at the MICS frequency band used in [50,51] are shown in Figure 10a,b, respectively. As shown in Figure 10, two layers of substrate were inserted over the ground by putting a radiating patch in the middle and a superstrate layer placed over the final radiating patch. Consequently, the implant antenna dimension miniaturized to π × (7.5) 2 × 1.9 mm^3^ [51] and 10 × 10 × 2.01 mm^3^ [50], respectively. Besides, some other stack patch layers implant antennas are given in [52,53,54,55].

#### 4.1.3. Insertion of Shorting Pin-In Wireless Technology 

**Figure 11:**
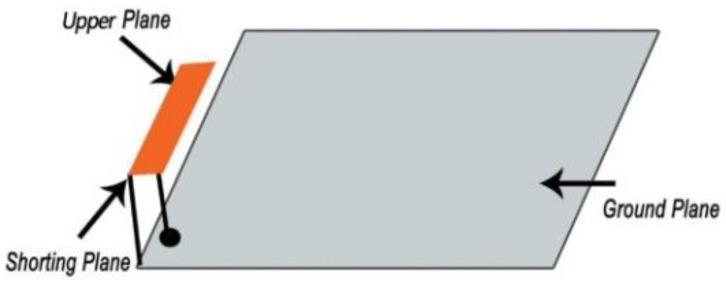
Typical shorting pin Planar Inverted F-Antenna (PIFA) antenna [57].

The Planar Inverted F-Antenna (PIFA) is a common example of a shorting pin antenna design technique. According to the PIFA theory, the shorting pin with antenna ground plan alter antenna resonance frequency from λ/2 to λ/4. Spiral slot shape shorting pin antenna was employed in [56], where the antenna dimension was miniaturized to 149.6 mm^3^ compared with other antennas reported. Figure 11 illustrates a typical shorting pin PIFA antenna deployed in [57]. Besides, several miniaturized PIFA antennas were reported in [58,59,60,61,62,63,64]. 

#### 4.1.4. Gain and Efficiency Enlargement Technique 

The absorption of radio wave frequency through the inhomogeneous lossy human body results in lower radiation efficiency. A significant amount of absorption happens due to the coupling with lossy tissue in the near reactive field. Recently, some implant antennas have been developed to solve low gain and efficiency problems. This section discusses some potential gain and efficiency enhancement techniques.

- Insulating Layers: A theoretical calculation has been made in [65], where a multilayered insulation model considering fat and dry skin was taken as standard. Notably, the insulation with zirconia and 4 mm thickness around the implant antenna gave the lowest attenuation of 34.5 dB than the other insulation materials (e.g., alumina, polyamide, peek, polypropylene). This theoretical calculation agreed with the experimental results reported in [11]. Besides, several materials-based insulation models were investigated in [66,67,68,69,70,71] and the results showed that insulation for biocompatibility reduces the attenuation, while increasing gain and radiation efficiency.
- Complimentary Split Ring Resonators (CSRRs) Antenna Model: The CSRR antenna model is an effective solution to enhance radiation efficiency and gain. This CSRR model compensates inductivity and electric field coupling with the near field due to the antenna’s negative permittivity [72,73]. The SAR is also reduced, which improves the radiation efficiency and gain. The CSRR implant antenna model for multiband (MICS, ISM, and 2.4 GHz) applications was designed and simulated in [74]. The simulation results showed that the electric field was at 403 MHz It can be noted that electric field absorption is reduced for the CSRR model compared to the non-CSRR model. Hence, radiation efficiency and gain are increased for all operational frequencies.

#### 4.1.5. Bandwidth Enhancement Technique

**Figure 12:**
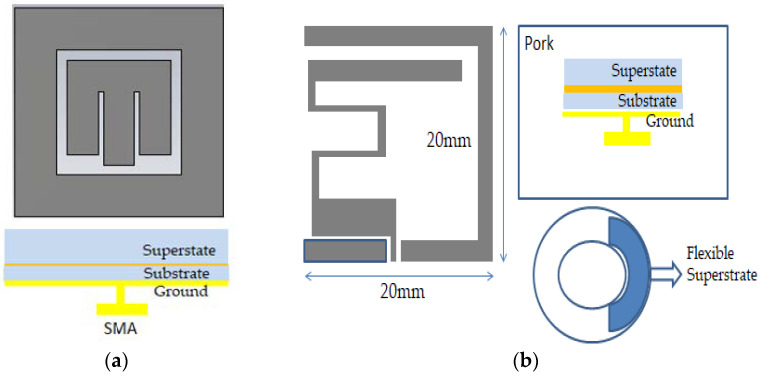
Bandwidth enhancement (BW) technique, (**a**) loop shape parasitic patch [75], (**b**) flexible antenna [76].

Deployment of wideband implant antennas in the medical frequency spectrum for biomedical telemetry applications is a research challenge. Typically, a parasitic patch is used as a traditional bandwidth enhancement (BW) technique [75], where a multi resonance mode was attained for square shape parasitic patches outside the main radiating patch. Hence, the bandwidth can be enhanced to 2.24–2.59 GHz using the multi resonance mode. Figure 12a illustrates the bandwidth enhancement technique via a loop shape parasitic patch [75], while Figure 12b shows a flexible structure bandwidth enhancement technique [76]. Because of the flexible structure, the antenna generated two coupling radiating patch structures with the ground. Hence, these two coupling structures attained 68% bandwidth applicable for the MICS frequency band. 

#### 4.1.6. Tuning Permanency Technique

The power absorption caused by the lossy environment around the implant antenna results in inefficient antenna operation. The implanted antenna is referred to as efficient when the operating frequency is tuned in a specific band area with efficient radiation efficiency. Even if antenna fundamentals state that the minimum antenna efficiency for 5G applications is 70%, it might vary depending on the appropriate implant applications. Recently, antenna designers have developed some techniques for tuning and impedance matching stability.

Planar antennas with a ground plane provide better tuning stability than antennas without a ground plane, such as loop and dipole antennas [77,78]. Besides, narrow operational bandwidth with a high superstrate biocompatibility system reduces the coupling with lossy tissue around the antenna, which leads to tuning stability and high radiation efficiency [77]. It is also found that the tuning of resonance frequency can be avoided if the antenna is operated on a wide frequency band [79,80].

### 4.2. Implant Antennas for Different Bio-Telemetry Applications

In this section, systems with implantable antennas for several biotelemetry applications, such as inside the human skull to monitor cerebrospinal fluid monitoring, blood pressure, and glucose level monitoring, will be summarized.

#### 4.2.1. Implant Antenna for Monitoring of the Healing of Bone Fracture

The healing process of a fractured bone is a problematic biological process, and it requires continuous observation with mechanical support for the re-establishment of a fractured bone in its natural condition. The fractured bone restoration process varies between several weeks depending on age, the number of fractures, and bone location [81]. An implantable antenna with a bone healing mechanical system would be an excellent solution for continuous observation of fractured bones. This sub-section summarizes the implant antennas used for the robust bone restoration process. 

**Figure 13:**
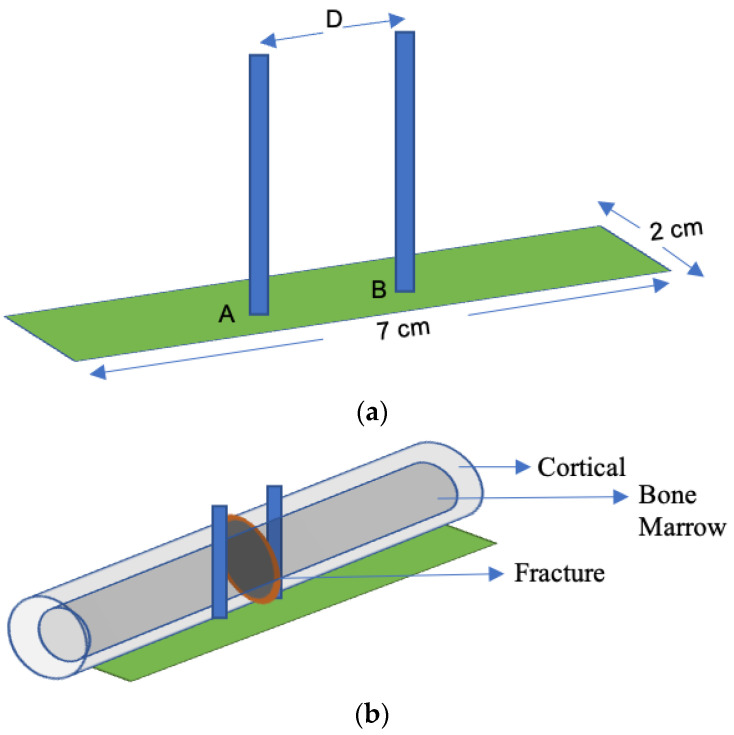
Implant antenna system for bone fracture restoration process. (**a**) Proposed monopole antenna, (**b**) monopole antenna position in artificial fracture bone phantom environment [81].

**Figure 14:**
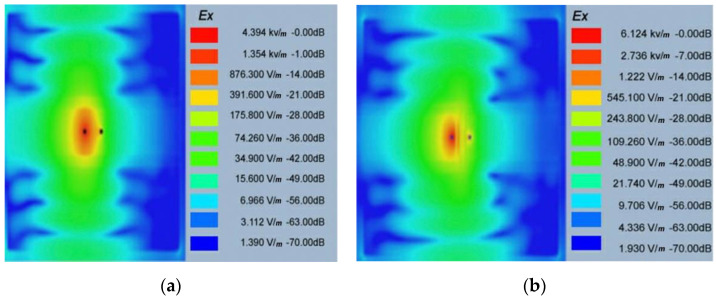
Electric field distribution at 1.8 GHz for 0% bone damage (**a**) and 100% fractured bone damage (**b**) [81].

In [81], an implant antenna system with two monopole antennas attached with a metal plate was proposed to observe the fractured bone restoration process. An artificial fracture bone phantom system where two monopole antennas were placed on both sides of the fracture is shown in Figure 13. The restoration process of a fractured bone was detected via the electric field distribution of the monopole antenna. Gabriel et al. [82] showed that relative permittivity and conductivity of blood are significantly higher than the bones across the radio frequency spectrum. It was mentioned that the higher conductivity and relative permittivity increase penetration loss, which reduces the electric field distribution of the propagation medium. In [81], the authors pointed out that the electric field distribution is substantially high when the bone damage is 0% compared with a fractured bone as shown in Figure 14. According to [83] the attenuation of the electric field in the Fresnel region is dependent on wave-number k for lossy medium, as stated by Equation (14). The wave-number k rely on relative permittivity $\varepsilon_{r}$ and conductivity σ as stated by Equation (15). Hence, higher values of the relative permittivity ($\varepsilon_{\mathrm{rblood}}=58.1,\;\varepsilon_{\mathrm{rbone}}=11.3$) and conductivity (σ_blood_ = 2.6, σ_bone_ = 0.4) in blood than bone constitutes a greater wave-number k for the fractured bone area, which leads to higher attenuation of the electric field in the fractured bone area due to the existence of blood. In conclusion, the reason behind the diversity of current distribution is due to the existence of blood in the bone fracture area, where there is no blood existence inside the restored fractured bone.

(14) $${\mathrm{Electric}\;\mathrm{field}}_{\mathrm{Attenuation}}=e^{-j\;\mathrm{kr}}/r$$

(15) $$K=\omega \sqrt{\mu_{0}\mu_{r}\varepsilon_{0}\left(\varepsilon_{r}-j\frac{\sigma }{\omega \varepsilon_{0}}\right)}$$

**Figure 15:**
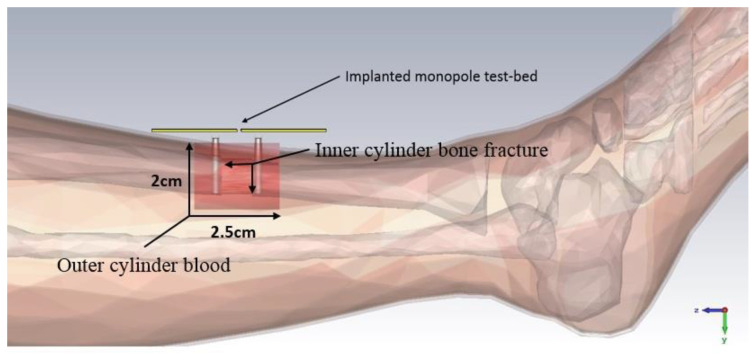
Simulating environment of monopole antenna in a fractured bone [20].

**Figure 16:**
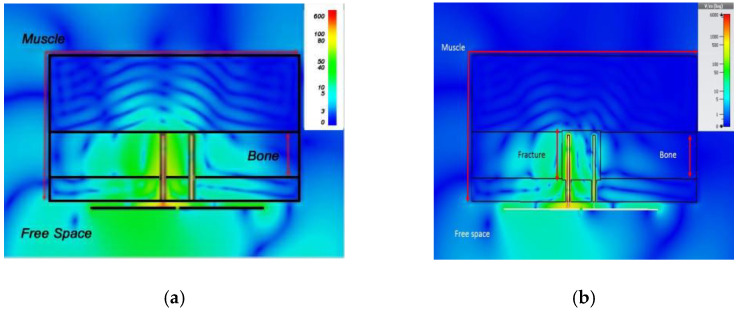
Electric field distribution at 2.5 GHz: during 0% bone damage period (**a**), fractured bone period (**b**) [20].

A dual monopole antenna was proposed in [20] for monitoring the fracture of the bone, which was simulated with a voxel model of a 26-year-old female on CST Microwave Studio virtual version as shown in Figure 15. The restoration process of fractured bone was monitored by a monopole antenna placed on both sides of the fracture and determined the bone condition by transmitting power from one monopole to another monopole. It can be observed in Figure 16 that during the 0% bone damage period a widely distributed electric field was obtained, while the electric field distribution was consolidated in the recovery phase. The fabrication of the antenna system and observation of monopole antenna performance inside the bone fracture will be briefly discussed later. 

#### 4.2.2. Implant Antenna for Glucose Level Monitoring in Blood

Glucose level monitoring in human blood plays an essential role in diagnosing life-threatening diseases (e.g., diabetes and hypoglycemia). Typically, chronic diabetic patients need to check blood samples daily to monitor glucose levels in the blood. The current method to check glucose levels is invasive and painful. Recently, implantable biosensors were developed to monitor glucose levels in the blood, which can provide a regular update about the glucose level of the diabetes patient. 

**Figure 17:**
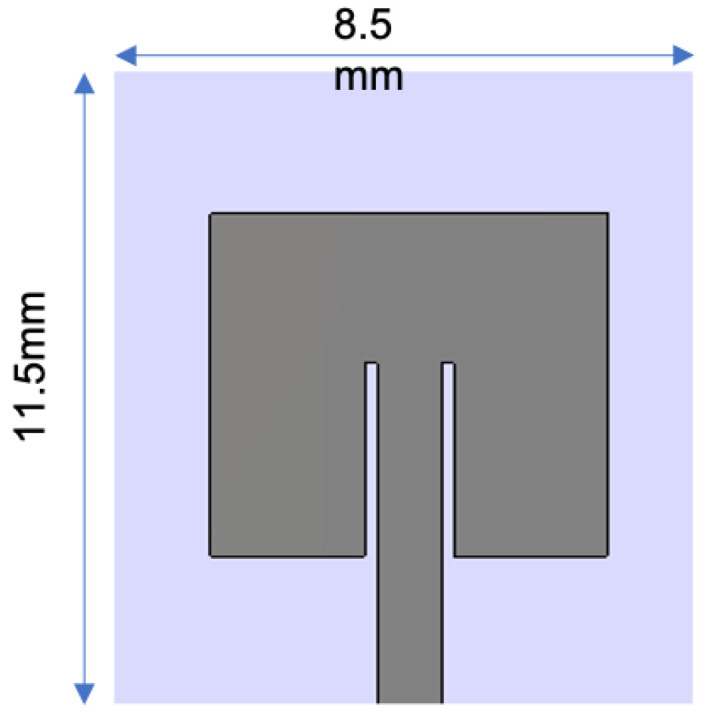
The glucose monitoring system in blood: geometry of implant antenna [84].

An implantable bio-sensor system to monitor the glucose level in blood was implemented using a frequency shift mechanism [84]. The implantable antenna geometry, return loss, and resonance frequency shift of the implant antenna with the increment of glucose level in blood are shown in Figure 17. Furthermore, it is noted that the implant antenna obtained 40 MHz frequency shifting for glucose level fluctuation in blood between (120–530) mg/dL on blood mimicking phantoms and 26 MHz frequency shifting for glucose level fluctuation in pig blood between (67–490) mg/dL.

#### 4.2.3. Implant Antenna for Diagnosing Brain Diseases

**Figure 18:**
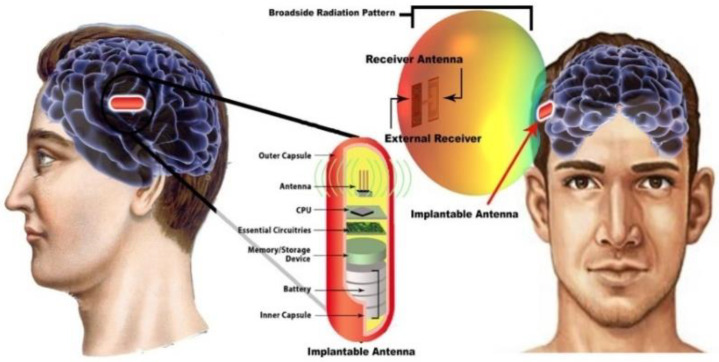
The basic mechanism of wireless brain monitoring system [85].

In diagnosing brain diseases and neurological disorders during therapy, an implantable wireless device and antenna will be a marvelous solution as upcoming medical technology. Nowadays, an implanted antenna is being used for restoring memory loss [67] and primary exposure of epileptic seizures. The basic mechanism of the wireless brain monitoring system is illustrated in Figure 18, where the implant antenna system contains a neural recorder that is connected with the transmitter system.

**Figure 19:**
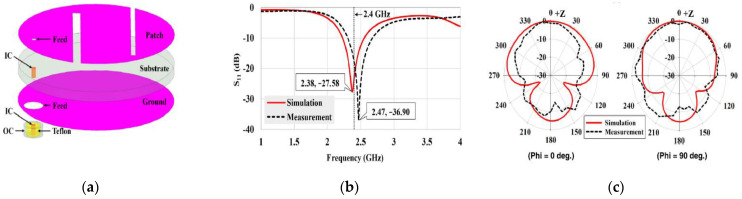
Geometry of implant antenna (**a**), antenna returns loss (**b**) and 2D radiation pattern (**c**) [85].

S. Hout and J. Chung in [85] reported a miniaturized circular shape implant antenna and the antenna was placed inside a seven-layer brain phantom as shown in Figure 18. Typically using a substrate with higher permittivity for miniaturizing antenna geometry results in a narrow bandwidth and lower radiation efficiency due to a high-quality factor [85]. Both of the substrate and biocompatible element with lower permittivity and dielectric constant substrate Taconic RF 3.5 ($\in_{r}\;=3.5,\;\mathrm{Tan}\delta =0.0018$) were used to obtain a 14.9% increase in bandwidth compared to the previous implant antenna at 2.4 GHz shown in Figure 19b. However, omnidirectional radiation patterns can cause harmful side effects inside brain tissue due to the frequency dependence characteristic of human tissue [86]. To ensure patient safety, the implant antenna proposed in [85] achieved a radiation pattern in an optimistic direction shown in Figure 19c. The outside antenna receiver can efficiently communicate with the inside implant antenna. Finally, the antenna was fabricated and validated by injecting inside seven layers of artificial tissue emulating (ATE) materials brain phantom in an in vitro experiment, where the measurement agreed with the simulation results.

#### 4.2.4. Implant Antenna for Blood Pressure Measurement

**Figure 20:**
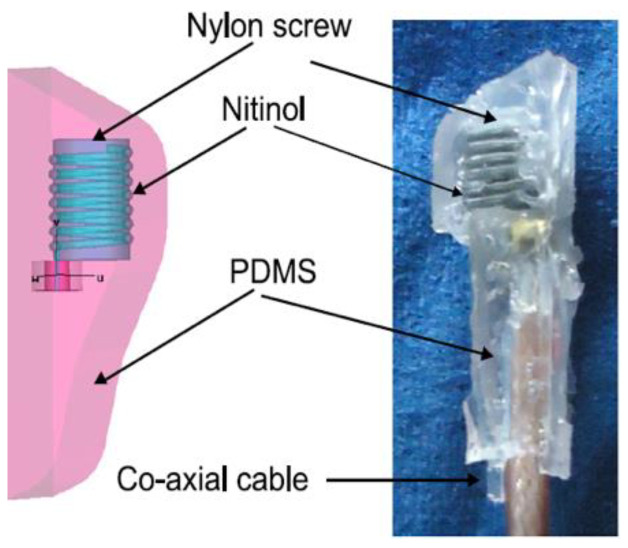
Snapshot of implant antenna for blood pressure measurement [21].

Frequent fall and rise of blood pressure can cause a stroke or severe cardiovascular disease for patients, and thus an accurate blood pressure measurement is critical to help medical personnel in managing several diseases that are related to blood pressure. Blood pressure measurement using an implantable antenna system inserted into the heart will be an excellent solution for heart patient monitoring. In [21], a pseudo normal mode helical antenna insulated by poly-di-methyl-siloxane (PDMS) layer is implemented and tested (Figure 20). The sensor and the implant antenna were put inside the left ventricle and experimented with a pig.

## 5. Fabrication and Implantation Process of the Implant Antenna 

Implantation of the implant antenna system is not only limited to antenna fabrication but also requires the design of a power management sub-system. The implementation of each part is carried out by complex procedures due to the inhomogeneity of the human body structure.

### 5.1. Implant Antenna Fabrication Process 

Implant antennas are typically simulated first before moving to the fabrication stage. The simulated antenna needs to be validated through the fabrication process. Since the fabrication of the simulated antenna is a vital part of any antenna design. Generally, the antenna fabrication process went through two stages: laboratory and industrial fabrication. Once the antenna fabrication results experimented in the laboratory match with the simulated result, it can be moved to the industrial production level.

#### 5.1.1. Antenna Fabrication 

Detailed methodology of the fabrication of the implant antenna has been adopted from [54]. The fabrication process follows the following steps.

**Figure 21:**
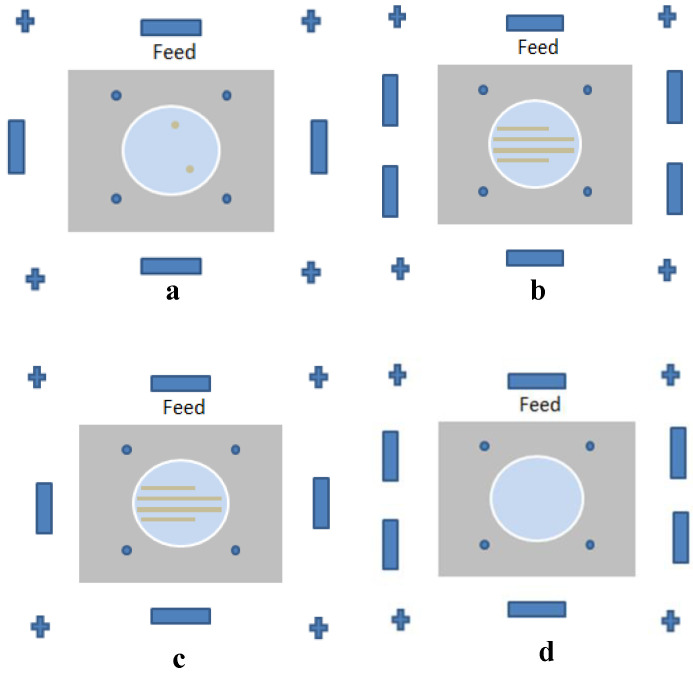
Photolithography process: (**a**) ground plane, (**b**) lower patch, (**c**) upper patch, and (**d**) superstrate [54].

A photolithography mask is a tool made to view the antenna geometry, and the antenna layer is stacked on its plane (Figure 21). The antenna layers are etched according to the antenna geometry via a photolithography mask. The lower substrate contains the ground and lower patch, the upper substrate contains the upper patch, and the superstrate is on the top. A circular hole is etched according to the patch geometry, where four pins are at the base of the mask. All layers were cut to the circular format and placed the antenna layers in a straight line. This process should be done without giving much more mechanical stress to the antenna. Finally, the antenna layers are aligned in a mountain format and glued to attach all layers. The shorting pin is connected with the ground plan and lower patch. Hence, the outer conductor of the co-axial feeding point is connected to the ground, while the inner conductor is soldered to the lower patch and upper patch. 

Following the validation of the antenna simulation result via the laboratory fabrication method, the industrial fabrication method is recommended for production. Low-temperature ceramic Co-fire (LTCC) antenna fabrication technology is a popular industrial fabrication method [32]. 

#### 5.1.2. Verification of Implant Antenna in Biological Environment 

The implantable antenna, which is fabricated using laboratory and industrial methods, needs to be verified in the biological environment. Depending on the type of experimental procedures, the verification process is classified into in-vitro and in-vivo processes. These two testing processes are described below.

**Figure 22:**
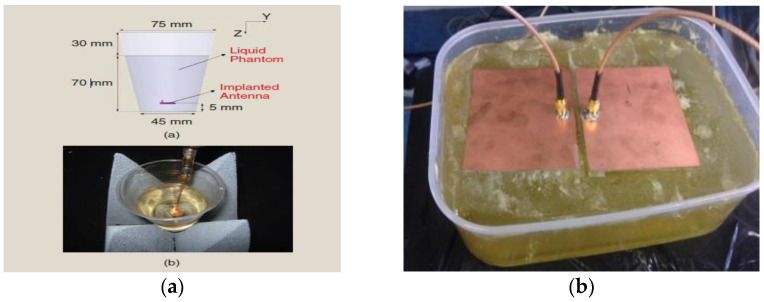
Implant antenna immersed in the artificial tissue environment, (**a**) [90] and three-layer phantom material (**b**) [20].

**Figure 23:**
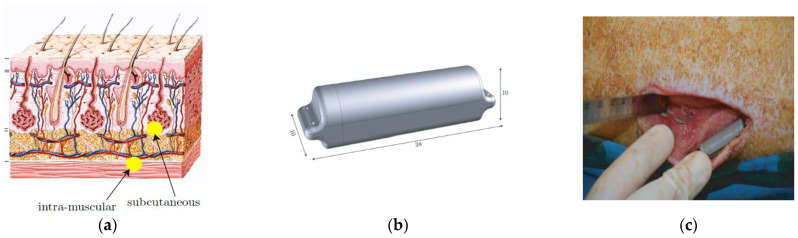
Cross-section view of pork body (**a**), biocompatible casing for implant antenna system (**b**), and injection of implant antenna inside the pork body (**c**) [10].

**Figure 24:**
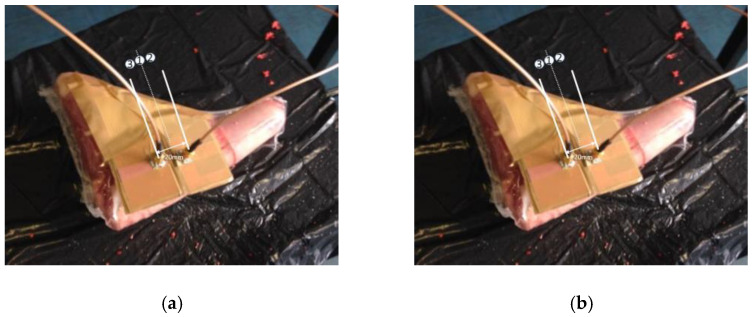
Implant antenna in vivo testing for monitoring the bone fracture healing (**a**) [20], and monitoring of blood pressure inside left ventricle (**b**) [21].

- In vitro Antenna Testing: The fabricated implant antenna is verified in the in vitro antenna testing process using an artificial biological environment [32,54]. Investigation of artificial emulation of the biological tissue environment in the MICS band has been performed in several recent works [64,87,88]. Gels are preferred rather than regular liquid for creating artificial tissue layers, where gels make the layers more solid. Deionized distilled water is used to make the synthetic tissue layers. An artificial biological environment containing 41.48% distilled water, 56.18% glucose, and 2.33% salt with a relative permittivity and conductivity of 46.7 and 0.69 s/m, respectively [87,89]. The properties of artificial phantoms are investigated using an Agilent network analyzer. The implant antenna prototype immersed in the artificial tissue environment is shown in Figure 22a [90]. It can be noted that the implant antenna is immersed in liquid at a distance, d from the surface. The prototype antenna is connected with a vector network analyzer (VNA) via co-axial feeding cable. An implant monopole antenna inserted in the three-layer materials phantoms, where two layers are of bone and one layer of muscle. The phantom is made up of six minerals consisting of flour, oil, deionized water, food color, sugar, and detergent. The in-vitro performance of the implant antenna was compared with the simulated antenna.
- In vivo Antenna Testing: In-vitro study carried out in an artificial biological environment cannot ensure the stability of the implanted antenna system, due to the lack of dynamic representation of a real biological environment in the in vitro study [30]. Hence, antenna testing in a real biological environment is highly recommended after in vitro testing. Before implantation of the prototype antenna inside the body, this must experience the temperature testing process below 100 °C. Generally, the implant antenna itself produces heat up to 60 °C temperature because of the battery and internal system components. Besides, the implant antenna is insulated by biocompatible material for protecting the antenna system from coupling loss. Figure 23 shows the in vivo experimental set-up, where pork body is taken as the standard environment, while Figure 24 a,b illustrates the in-vivo testing of the bone fracture healing process and monitoring of blood pressure inside the left ventricle respectively.

The outside base station [10] must communicate with the implant antenna inside the body and process information from the implant antenna towards the database station. In [10], a base station system was shown to consist of four segments: Zarlink antenna module, image acquisition system, and a USB modem with a computer.

### 5.2. Power Management

**Figure 25:**
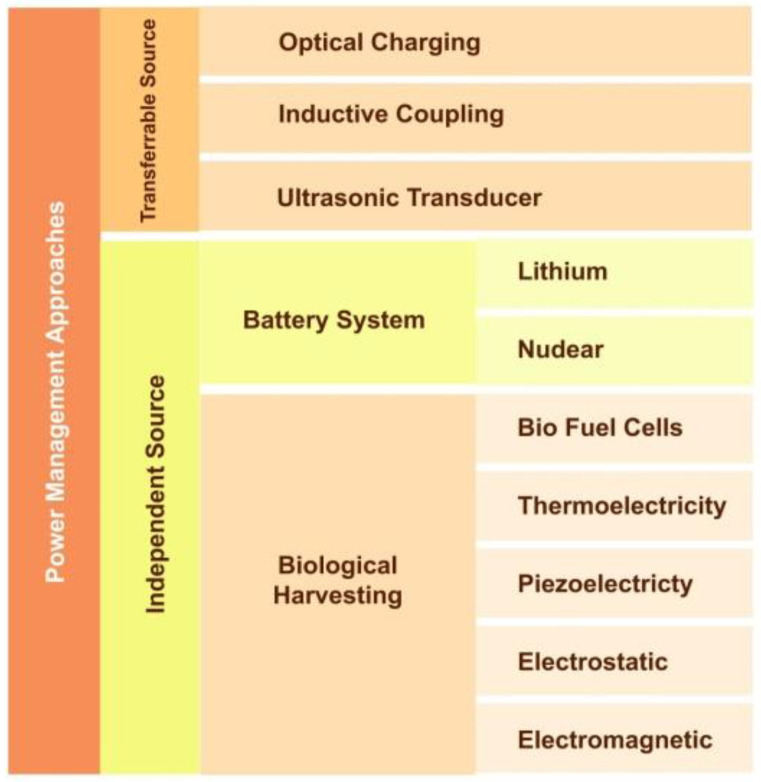
Classification of implant antenna power management techniques.

The power management system of the implant antenna system is an ongoing research challenge. Efficient power management of the implant antenna system is still challenging even with the rapid development of the applications of implants. There are two major types of power sources for implants: an independent source and a power transmission mechanism (Figure 25).

#### 5.2.1. Independent Power Approaches

Independent power approaches referred to an active power source attached to the implant antenna system. The Independent power sources for implant antennas are of two types: battery and biological harvesting.

Although the battery system has some disadvantages, two different batteries (lithium and nuclear battery) have been used in implant medical devices. Lithium batteries have been safely used for implantable medical devices (IMDs) such as cochlear implants and pacemakers [12]. The nuclear battery would be a great power source in the implant antenna system, where nuclear energy is transformed into electricity emitted by the radioisotope. Consequently, the nuclear battery source can provide a much longer lifespan (10 years) than any other power source. A microwatt level nuclear beta-cell with dimension 1.02 × 1.52 cm^2^ was launched by Medtronics USA, which generated 50 μW of power [91]. The stability of the power supplied by the nuclear source is extraordinarily high and is not influenced by the biological environment (temperature, pressure, and electric field). Therefore, it is a potential power source for biomedical implants in the near future.

Biological energy harvesting is defined as the battery-less power management system that depends on biological activities to generate power. Several systems have been utilized for energy harvesting, such as biofuel cells, thermoelectricity, piezoelectricity, electrostatic, and electromagnetic systems.

**Figure 26:**
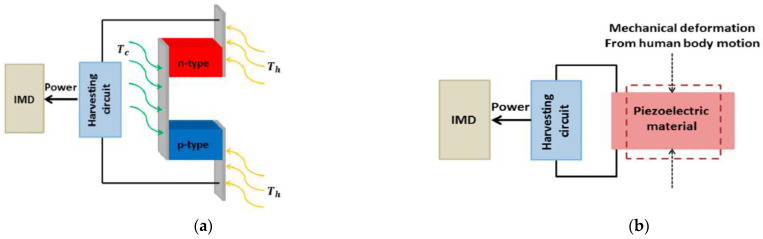

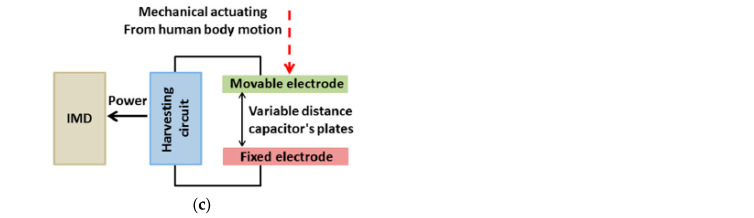
Energy harvesting using a thermoelectric mechanism (**a**), the piezoelectric mechanism (**b**), and the electrostatic system (**c**) [12].

A biofuel cell is a method to produce electricity from biochemical energy generated through electrochemical reactions with biological substances. A miniaturized bio-fuel was introduced in 2003, which was able to produce 2.4 μW of 0.52 V inside a grape [92]. Some other bio-fuel cells were investigated for the application of IMD since 1970 [93]. Thermoelectricity is defined as the generation of electrical energy from the temperature difference mechanism inside the human body. Naturally, the human body represents an inhomogeneous temperature environment, and when the temperature difference is the process through a thermoelectric module, and then a potential difference emerges, which generates electricity (Figure 26a). Thermoelectricity-based power sources were employed in [94,95], where the electricity produced 5.8 μW/0.19 cm^2^ [94] and 180 μW/cm^2^ [95] by thermoelectric effect in the biological environment, which is sufficient for implant medical devices. 

Electricity can be generated by piezoelectric mechanisms. Due to the motion of the human body, electricity can be harvested. Typically, the motion of the human body is classified into two categories: continuous motion such as human respiration and blood flow; and discrete motion such as walking and organ movement. The maximum amount of energy that can be produced by discrete organs’ motion is up to 69.8 W. On the other hand, continuous motions generate lower electricity [96]. As stated in [97], continuous blood flow can produce only 33 μW. Figure 26b shows the mechanism of generating electric power using the piezoelectric technique [12].

The electrostatic generators produce electrical power by utilizing electrostatic induction due to human body motion and electrode movement. The typical electrostatic system is shown in Figure 26c, where two electrodes are kept at a distance with the vacuum space, and the electricity produced when electrodes move due to the human body [98]. In recent days, research has been done on electrostatic generators to apply the implant antenna system [99].

#### 5.2.2. Transferrable Source

Although the independent power system attached to the implant system can avoid the issue of multiple battery replacements, the low output power and the possibility of toxicity from the power system make the system inefficient. Researchers focus on transferrable energy through the human body tissue to implant antenna systems to meet this research challenge. Different techniques have been discussed in the literature to transfer power to implantable systems.

**Figure 27:**
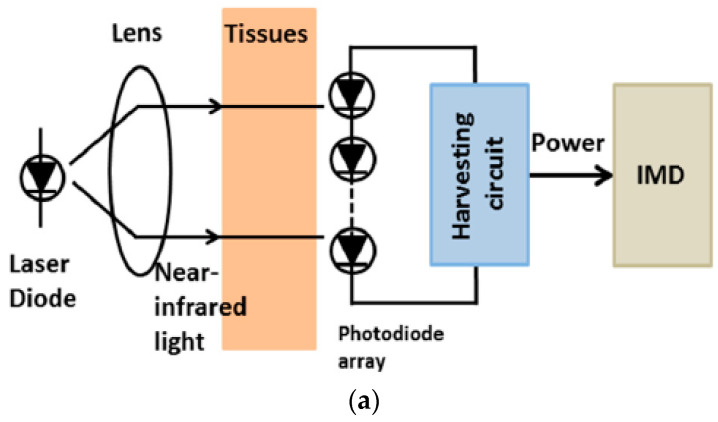

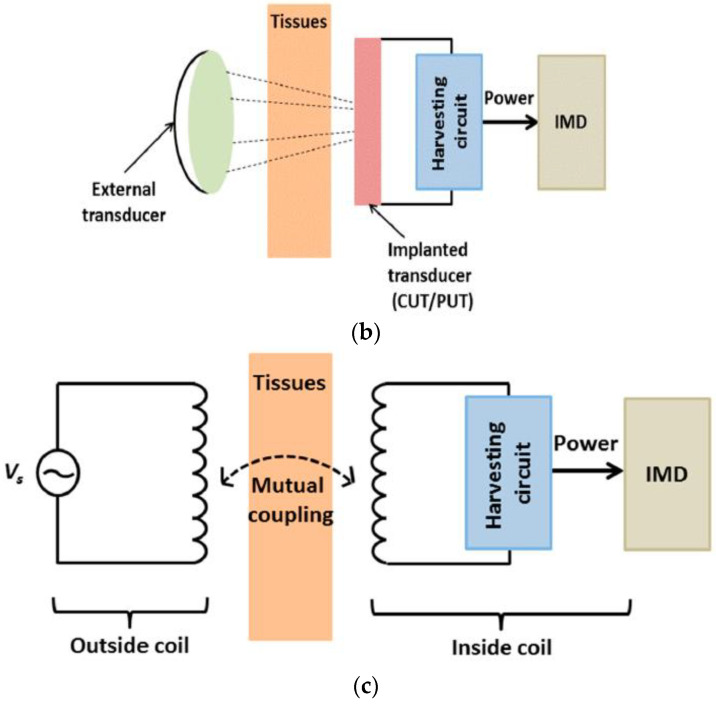
Block diagram of (**a**) optical charging, (**b**) ultrasonic transducer, (**c**) inductive coupling [12].

Optical charging is defined as a technique to charge the photovoltaic cells inside the implant system by the infrared laser diode from the external body unit to transfer power. The photovoltaic cells convert light energy into electrical power [13,14]. A typical mechanism of optical charging is shown in Figure 27a. Optical charging was deployed in [15], where the photovoltaic cells covering area 2.1 cm^2^ were used to generate 22 mW/cm^2^, which is sufficient for operating a pacemaker for 24 h.

An ultrasonic transducer produces an ultrasonic wave produced by an external mechanical process that penetrates the tissues and excited the implanted capacitive ultrasonic transducer (CUT) or piezoelectric ultrasonic transducer (PUT). Hence, the acoustic wave received by an implanted transducer is converted into electrical power [16,100] (Figure 22b). In [101], a piezoelectric transducer of 3.5 mm diameter implanted inside living tissue was shown to generate 1.5 mW/cm^2^ for micro-stimulator applications. 

The power transfer through the induction coupling process can be a possible solution for biotelemetry of the medical systems. The induction coupling mechanism is shown in Figure 22, where electromagnetic voltage is induced in the coil of the implanted antenna via mutual coupling through biological tissue layers. In [102], the induction coupling system was employed for implant medical devices inside the rabbit and a coil of 10 mm diameter was used to generate 19 mW at a carrier frequency of 10 MHz. However, the power transfer efficiency to the implanted device inside the body depends on several factors including alignment, distance, and coupling match between the coils.

## 6. Limitations of the Current Implantable Antenna System and Imminent Research Challenges

Applications of implantable medical devices (IMDs) in the medical sector have been increasing tremendously. Nevertheless, the functioning and implantation difficulties of IMDs make them inefficient. This section will identify the limitations of the implant antenna system and discusses potential research challenges:

▪ Absorption of an electromagnetic wave happens due to coupling with lossy tissue in the near reactive and far-field. A significant amount of absorption results in low radiation efficiency and inefficient antenna operation. The radiating wave travels through the near field and far field to reach the outside receiver antenna, since the absorption in the far-field is unavoidable. However, the absorption of a radio wave can be avoided if the biocompatibility of the implant antenna is covered near the field. Therefore, implant antenna design with biocompatibility covering the near field will be a possible research challenge in the future.
▪ Inhomogeneous biological tissues and organs form the human body. Besides, the dimension and characteristics of biological tissues vary from time to time and gender to gender. Therefore, the detuning effect is considered the primary research challenge in designing an implanted antenna for biotelemetry applications. The implantable antenna tuned in a resonance frequency may not be stable for other persons or locations. In research work [103], the detuning effect was performed on four anatomical bodies and thirty tissues. An almost 70 MHz frequency shift from the primary resonance frequency was observed. To date, implant antenna design and experiments are limited to only a single tissue environment, which will be a significant disadvantage for diverse tissue environments. Hence, the upcoming implant antenna must be investigated in different biological environments for efficient antenna operation.
▪ Injection of a radio-frequency device inside the human body may have a long-term complex health problem due to radiative power absorption. Therefore, effective implant antenna design with limiting SAR value and appropriate selection of biocompatible materials will be the primary research concern.
▪ Conventional antenna dimension miniaturization techniques tend to narrow operational bandwidth. The narrowband operation cannot avoid the detuning effect that happens inside the biological environment. Even though biocompatible encapsulation of the implant antenna is utilized to enhance radiation efficiency and gain, it expands the overall thickness of the implant medical device. Hence, the antenna design technique with adequate operational bandwidth, radiation efficiency, and gain is still a challenging issue in implantable medical device technology.
▪ Generally, IMD inside the human body is powered by a battery system. Still, a limited life-time power system and the bulky dimension cause inadequacy in the implant antenna power system. Also, the replacement of the battery system via a surgical procedure makes implementation difficult. The nuclear battery system can be an effective solution to the limited battery life. The nuclear battery provides stable power of 50 μW over an extended range of duration [91]. The only challenge is providing a proper guideline to avoid the poisonousness of radioactive radiation.
▪ A biofuel cell is a battery-less power system that converts biochemical energy into electricity. The primary limitation of a biofuel cell is micro-watt level power, which limits its usefulness in IMDs. Moreover, biofuel cells can harm the tissue cell, even though it is biocompatible in laboratory testing [95].
▪ There are also some other battery-less power system for implant antennas. For example thermoelectricity, piezoelectricity, and electrostatic generators. Although those provide microwatt-level power over a long time, biocompatibility and proper design are the major research challenges of those power source models.
▪ Wireless power transfer is considered a promising solution in the implantable antenna power system [104]. Optical charging, ultrasonic transducers, and inductive coupling are the key examples of wireless power transfer in the implant antenna system to transfer milli-watt level power in the system. Nevertheless, during the power transfer with those systems, skin temperature rises, causing tissue damage and physical pain inside the body. Besides, there are limiting factors related to power transfer, which can reduce efficiency.

## 7. Summary

Significant progress has been made in medical implant technology due to the colossal demand for diagnosing life-threatening diseases. This paper describes an investigation of medical implant antenna technology and imminent research challenges. The major frequency spectrum allocation for medical implants lies within the sub-GHz range defined by FCC and ECC. This paper describes factors that affect radio wave propagation and implantable antenna geometry inside the human tissue environment by the fundamental wave propagation concept. The rise of conductivity, permittivity, and permeability of the hostile tissue environment affects radio wave propagation and affects the antenna structure. Moreover, this paper summarizes several existing medical implantable antenna design techniques and categorizes antennas according to the biotelemetry applications. Besides, this paper reviews the antenna fabrication process and illustrates antenna verification procedures including in vitro and in vivo testing, where an animal body environment is taken as a standard for the testing of implantable antennas. Furthermore, power systems for the implant antennas are summarized. Finally, the article concludes with the limitations of existing implant antenna technology and imminent research challenges in this area.

## Data Availability

Not applicable.
